# Repression of Interlayer Recombination by Graphene Generates a Sensitive Nanostructured 2D vdW Heterostructure Based Photodetector

**DOI:** 10.1002/advs.202100503

**Published:** 2021-05-20

**Authors:** Huide Wang, Shan Gao, Feng Zhang, Fanxu Meng, Zhinan Guo, Rui Cao, Yonghong Zeng, Jinlai Zhao, Si Chen, Haiguo Hu, Yu‐Jia Zeng, Sung Jin Kim, Dianyuan Fan, Han Zhang, Paras N. Prasad

**Affiliations:** ^1^ Institute of Microscale Optoelectronics International Collaborative Laboratory of 2D Materials for Optoelectronics Science and Technology College of Physics and Optoelectronic Engineering Guangdong Laboratory of Artificial Intelligence and Digital Economy (SZ) Shenzhen University Shenzhen 518060 China; ^2^ Department of Electrical and Computer Engineering University of Miami Coral Gables FL 33146 USA; ^3^ Institute for Lasers, Photonics, and Biophotonics and Department of Chemistry University at Buffalo The State University of New York Buffalo NY 14260 USA

**Keywords:** black phosphorus, graphene, interlayer recombination, sensitive photodetectors, vdW heterostructures

## Abstract

Great success in 2D van der Waals (vdW) heterostructures based photodetectors is obtained owing to the unique electronic and optoelectronic properties of 2D materials. Performance of photodetectors based 2D vdW heterojunctions at atomic scale is more sensitive to the nanointerface of the heterojunction than conventional bulk heterojunction. Here, a nanoengineered heterostructure for the first‐time demonstration of a nanointerface using an inserted graphene layer between black phosphorus (BP) and InSe which inhibits interlayer recombination and greatly improves photodetection performances is presented. In addition, a transition of the transport characteristics of the device is induced by graphene, from diffusion motion of minority carriers to drift motion of majority carriers. These two reasons together with an internal photoemission effect make the BP/G/InSe‐based photodetector have ultrahigh specific detectivity at room temperature. The results demonstrate that high‐performance vdW heterostructure photodetectors can be achieved through simple structural manipulation of the heterojunction interface on nanoscale.

## Graphical abstract

A nanoscale interface engineering in which few‐layer graphene (G) is inserted into the interlayer of black phosphorus (BP)/InSe, forming a 2D BP/G/InSe van der Waals heterojunction, is introduced. Effective electron transfer from BP to InSe is facilitated by the insertion of G, which induces a better photoresponse performance of the BP/G/InSe device.

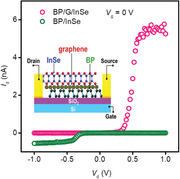

## 1 Introduction

Atomically thin 2D materials with nanoscale manipulable interfaces have drawn considerable attention on high‐performance photodetection applications, owing to their excellent mechanical, electrical, and optoelectronics properties.^[^ ^1^, ^2^, ^3^, ^4^, ^5^, ^6^, ^7^, ^8^, ^9^, ^10^, ^11^, ^12^ ^]^ In particular, layers of different 2D materials can be combined into 2D heterostructures by van der Waals (vdW) forces without considering lattice mismatching. This feature greatly expands the design strategy of 2D materials based optoelectronics devices for practical applications.^[^ ^13^, ^14^, ^15^, ^16^, ^17^, ^18^, ^19^ ^]^ It is worth mentioning that the performance of photodetectors based on 2D vdW heterojunctions at the atomic scale is more sensitive to the nanointerface of heterojunction than that of conventional bulk heterojunction. Interlayer recombinations (Coulomb interaction Langevin processes and traps‐assisted Shockley–Read–Hall [SRH] recombination) in p‐n type vdW heterojunctions such as WSe_2_/MoS_2_^[^ ^20^ ^]^ and GaTe/MoS_2_^[^ ^21^ ^]^ were observed at the nanoscale heterojunction interface, which is proportional to the concentration difference of majority carriers between the two materials, as indicated by the gate voltage regulating characteristics. When the concentration difference of majority carriers is large, SRH recombination became the main mechanism of recombination, which is originated from the defects/trap states at the interface of the heterojunctions. Therefore, the defects/trap states in 2D vdW heterojunctions are an important aspect to limit the further improvement of photodetection performance.

Recently, p‐type elemental 2D black phosphorus (BP) and its p‐n vdW heterojunction‐based photodetectors have been reported for broadband^[^ ^22^, ^23^ ^]^ and polarization‐sensitive^[^ ^24^, ^25^, ^26^ ^]^ photodetection. Ballistic avalanche phenomenon in BP/InSe vdW heterojunction^[^ ^15^ ^]^ has been discovered in 2019, showing its potential application in single‐photon detection. However, as a heavily doped p‐type semiconductor, the concentration of majority carrier^[^ ^26^ ^]^ (hole) of 2D BP is higher than 10^18^ cm^−3^. It is challenging to suppress the interlayer recombination caused by the large concentration difference of majority carriers in BP‐based vdW heterojunctions. Therefore, it needs other methods to protect the photogenerated carriers from the interlayer recombination (dominated by SRH recombination) for the purpose of better photoresponse performance, rather than reducing the concentration difference of the majority carrier at the heterojunctions. Few‐layer graphene (G) as a semi‐metallic 2D material, has lots of free electrons coming from the overlap of its conduction and valence bands.^[^ ^27^ ^]^ If few‐layer graphene has been inserted in a BP‐based heterojunction, the free electrons from graphene would fill the defects/trap states at the interface, which will effectively inhibit the interlayer recombination and reduce the loss of photogenerated carriers.

Here, we introduce a nanoscale interface engineering in which G has been inserted into the interlayer of BP/InSe, forming a 2D BP/G/InSe vdW nanoscale heterojunction. Effective electron transfer from BP through G to InSe was observed through transient absorption (TA) spectra, indicating that G can facilitate nanoscale electron transfer at the interface of BP/InSe, which inhibited the interlayer recombination coming from the imbalanced majority carrier concentration between BP and InSe. At the same time, owing to the effective transfer of electrons between the conduction bands of BP and InSe caused by the insertion of graphene and the high work function electrodes, the rectification characteristic of the device was transferred from the p‐n junction between BP and InSe to the Schottky junction between Au and InSe. And the carrier transport mechanism in the device was transformed from the diffusion motion of minority carrier to the drift motion of majority carrier. These two reasons, together with the internal photoemission (IPE) effect^[^ ^28^ ^]^ in the device, make the 2D BP/G/InSe vdW sandwiched heterojunction based photodetector exhibit significantly improved performance with response wavelength range of 405–2000 nm and D* as high as 3.19 × 10^15^ Jones at 532 nm and 3.44 × 10^14^ Jones at 2000 nm. Such high D* of the device at 2000 nm shows its great application on sensitive near‐infrared detection.

## 2 Results and Discussion

**Figure 1:**
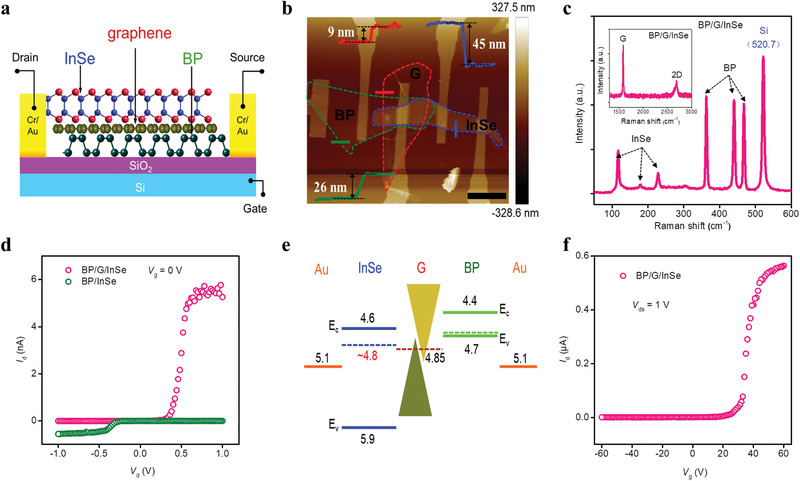
Structure and transport behavior of BP/G/InSe vdW heterojunction. a) Schematic of a cross‐sectional structure of the BP/G/InSe photodetector (Drain: drain voltage; Source: source voltage; Gate: backgate voltage). Drain and source electrodes contact InSe and BP, respectively. b) AFM image of the device. Scale bar, 10  µm. c) Raman spectra of the overlapped parts of BP, G, and InSe of the device. d) Output curves of BP/InSe and BP/G/InSe devices show the rectifying direction of BP/G/InSe is reversed after insertion of G, and its forward current is increased. e) Energy band diagram of BP, G, and InSe before contact. *E*_c_, *E*_v_, and the corresponding numbers represent minimum values of the conduction band and maximum values of the valence band of the semiconductors. Dotted lines represent Fermi levels of BP, G, and InSe. f) Transfer curve of the BP/G/InSe device. It shows that the BP/G/InSe device is an n‐type transport device, which is the same as InSe device.

We fabricated such a BP/G/InSe heterostructure‐based photodetector by exfoliating layers from the bulk forms of the respective materials and building a vertical heterostructure on nanoelectrodes using a 2D transfer technique. The drain and source electrodes are in contact with InSe and BP, respectively. Current is generated by a drain–source voltage (*V*_ds_) applied across the two electrodes flowing through the BP/G/InSe channel (**Figure** **1**a), which can be modulated by the backgate voltage (*V*_g_) applied to the p^+^‐doped silicon substrate. Optical microscopy (Figure S1, Supporting Information) and atomic force microscopy (AFM) (Figure 1b) of the heterostructure show the thickness of the BP, graphene, and InSe sheet is ≈26 nm, ≈9 nm, and ≈45 nm, respectively. Raman spectra of the BP/G/InSe heterojunction on a SiO_2_/Si substrate shown in Figure 1c contain six characteristic peaks from BP and InSe^[^ ^29^, ^30^ ^]^ and two peaks at ≈1580 and 2700 cm^−1^ from few‐layer graphene.^[^ ^31^, ^32^ ^]^

Compared to the BP/InSe device, insertion of a few‐layer (nanoscale thick) graphene between the BP and InSe sheets reversed the rectifying direction and increased the forward current of the BP/G/InSe device (Figure 1d). To understand this phenomenon, we used Kelvin probe force microscopy (KPFM) to obtain the positions of the Fermi energy levels of BP, G, and InSe (Figure S2, Supporting Information) and analyzed the energy band diagram of the two heterojunction devices (Figure 1e). From the energy band diagram, the BP/InSe control device is clearly a typical p‐n junction device (Figure S3, Supporting Information). However, after inserting graphene, G/BP/Au forms a quasi‐ohmic contact with InSe, allowing electrons to pass through the BP/Au interface by tunneling effect. This causes the built‐in electric field of the device to move from the original BP/InSe interface to the Au/InSe interface, turning the device into a Schottky device (Figures S4 and S5, Supporting Information). When the high work function Au electrodes are replaced with chromium (Cr) electrodes, no rectification occurs, demonstrating that the Schottky junction is also necessary for this transition (Figure S6, Supporting Information). The same n‐type transport properties of the BP/G/InSe junction shown in Figure 1f and InSe shown in Figure S7 (Supporting Information) further support this transformation. The conversion from a p‐n to a Schottky junction changes the overall transport property of the device from diffusion motion of minority carrier (p‐n junction) to drift motion of majority carrier (Schottky junction). This causes the maximum value of the forward current (*V*_d_ = 1 V) in the BP/G/InSe device to be larger than that in the control BP/InSe device (*V*_d_ = −1 V) (Figure 1d). The ideality factors (IF) of the BP/InSe and BP/G/InSe devices were obtained based on their *I*–*V* curves according to the formula:^[^ ^21^ ^]^ $\mathrm{IF}\;=\;\frac{q}{k_{0}T}\;\frac{dV}{\mathrm{dln}(J)}$, where *q* is the electron charge, *k*_0_ is the Boltzmann constant, *T* is the temperature, *V* is the source–drain voltage, and *J* is the source–drain current density. The results show that the ideality factor of the BP/G/InSe device is 2.28, which is slightly smaller than the ideality factor of the BP/InSe device of 2.48, indicating that the insertion of G has inhibited the interlayer recombination current to a certain extent. The value of the IF here larger than 2 may be caused by the existence of In or Se vacancies in InSe.^[^ ^21^, ^33^ ^]^

**Figure 2:**
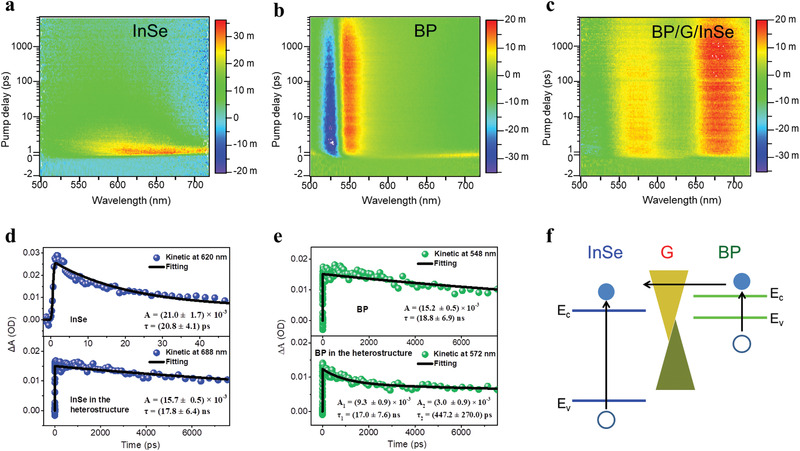
Interlayer charge transfer in BP/G/InSe heterojunctions. Transient absorption spectra of a) InSe, b) BP, and c) BP/G/InSe heterostructure. d) Test and fitting results of the PIA signal of InSe obtained from separate InSe and BP/G/InSe heterostructures. e) Test and fitting results of the PIA signal of BP obtained from separate BP and BP/G/InSe heterostructures. The carrier lifetime is fitted by single exponential function: Δ*A* = *A*exp(−*t*/*τ*) or biexponential function: Δ*A* = *A*_1_exp(−*t*/*τ*_1_) + *A*_2_exp(−*t*/*τ*_2_). The time constants are extracted to be *τ* = (17.8 ± 6.4) ns for InSe in the heterostructure, much longer than that in control InSe (*τ* = (20.8 ± 4.1) ps). On the other hand, the average time constants are extracted to be *τ*_ave_ ≈ 13.0 ns for BP in the heterostructure, which are lower than that in control BP (*τ* = (18.8 ± 6.9) ns). *τ*_ave_ is calculated by the equation: *τ*_ave_ = (*A*_1_ *τ*_1_ + *A*_2_ *τ*_2_)/(*A*_1_ + *A*_2_). f) Schematic diagram of charge transfer in the BP/G/InSe heterostructure.

To understand whether the increased *I*_d_–*V*_d_ forward current is because of graphene preventing carrier recombination between BP and InSe, we studied the excitation and recombination dynamics of BP, InSe, and the BP/G/InSe heterostructure using femtosecond‐resolved transient absorption (TA) micro‐spectroscopy (Figures S8 and S9, Supporting Information).^[^ ^34^, ^35^, ^36^ ^]^ TA spectra for InSe showed an ultrafast photoinduced absorption (PIA) between 570 and 720 nm (**Figure** **2**a) while BP displayed both PIA at 540–560 nm and photoinduced bleaching (PIB) at 520–530 nm (Figure 2b). Although the TA spectra of BP/G/InSe shown in Figure 2c can be regarded as the superposition of InSe and BP, the wavelength range of the PIA and PIB, and the carrier lifetime in the heterostructure were all dramatically different from the control InSe and BP. The carrier lifetime of the excited state electrons of InSe in the heterostructure lengthened from *τ* = (20.8 ± 4.1) ps to *τ* = (17.8 ± 6.4) ns, indicating that electrons from other materials (such as G and BP) were transferred into InSe (Figure 2d). Correspondingly, the carrier lifetime of the excited state electrons of BP in the heterostructure decreased from *τ* = (18.8 ± 6.9) ns to *τ*_ave_ ≈ 13.0 ns, showing an enhanced excited electron transfer to other materials, namely, G or InSe (Figure 2e). Compared with the control BP device, the PIB and PIA peak of BP in the BP/G/InSe heterostructure were both broadened, and a blue and redshift occurred, respectively, further implying electron transfer occurred and did so efficiently in the heterostructures^[^ ^37^ ^]^ (Figure 2f). Together, these results demonstrate that insertion of graphene transformed the structure of the heterojunction and inhibited interlayer recombination, which simultaneously increased the number of effective carriers in BP/G/InSe. These changes improved the transport properties of the device, making them highly suitable as photodetectors.

**Figure 3:**
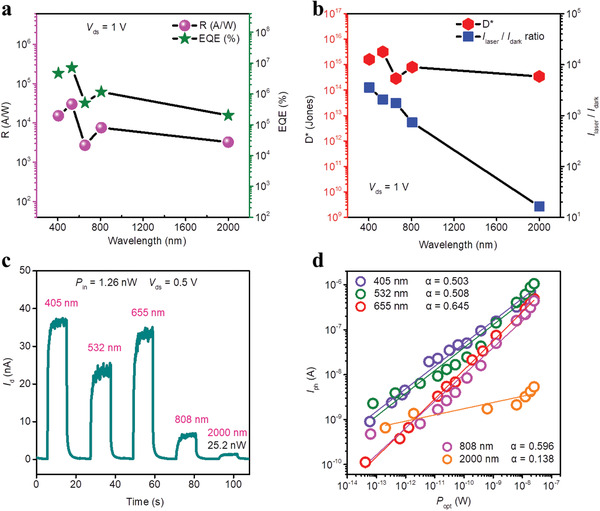
The photoresponse performance of the BP/G/InSe devices. a) Wavelength‐dependent *R* and EQE values under low illumination power. The laser power corresponding to 405, 532, 655, 808, and 2000 nm laser are 59.2, 75.6, 41.6, 63.0, and 202 fW, respectively. b) Red dots: *D* ^*^ curve for different wavelength laser illumination at *V*_ds_ = 1 V, *V*_g_ = 0 V. The laser power corresponding to 405, 532, 655, 808, and 2000 nm laser are 59.2, 75.6, 41.6, 63.0, and 202 fW, respectively. Blue dots: Light current/dark current ratio curve for different wavelength laser illumination at *V*_ds_ = 1 V, *V*_g_ = 0 V. The laser power corresponding to 405, 532, 655, 808, and 2000 nm are 18.9, 25.2, 25.2, 25.2, and 25.2 nW, respectively. c) Time dependence of light current and dark current curves under different wavelength laser illumination at *V*_ds_ = 0.5 V, *V*_g_ = 0 V. d) Photocurrents (*I*_ph_) under various light power (*P*_opt_) with different wavelength. The fitting lines give the relationships of *I*_ph_ ≈ *A* * *P*_opt_ *^*α*^* (where *A* is a constant) with *α* = 0.503, 0.508, 0.645, 0.596, and 0.138 for *λ* = 405, 532, 655, 808, and 2000 nm, respectively. *V*_ds_ = 1 V and *V*_gs_ = 0 V.

We further studied the photoresponse characteristics of the BP/G/InSe heterostructure device. Typical *I*_ds_–*V*_ds_ curves of the BP/G/InSe device in the dark and under different illumination powers at excitation wavelengths of 405, 532, 655, 808, and 2000 nm show the device has a broad spectral photoresponse even at a low power density of 0.1 µW cm^−2^ (**Figure** **3**a and Figure S10, Supporting Information). Higher laser power resulted in larger photocurrents in the device. When irradiated at 532 nm using low power (75.6 fW) light, the responsivity (*R*) of the device exceeded 3.02 × 10^4^ A W^−1^ (Figure S12, Supporting Information). Consistent with *R*, the external quantum efficiency (EQE) values are also very large, up to 7.04 × 10^6^%. In addition, the device has excellent photoresponse for 405 nm (Figure S11, Supporting Information), 655 nm (Figure S13, Supporting Information), 808 nm (Figure S14, Supporting Information), and 2000 nm (Figure S15, Supporting Information) light. Notably, under 2000 nm light (202 fW) irradiation, the value of *R* and EQE respectively reached 3.25 × 10^3^ A W^−1^ and 2.02 × 10^5^%, which is several orders of magnitude larger than previous BP‐based NIR photodetectors.^[^ ^14^, ^25^, ^38^ ^]^

The photoresponse of the BP/G/InSe device was further measured by calculating the *I*_laser_/*I*_dark_ ratio, a parameter directly related to specific detectivity (D*), which is the key index of photodetectors. The *I*_laser_/*I*_dark_ ratios of the BP/G/InSe device illuminated by 405 nm laser is higher than 3.5 × 10^3^, while the value corresponding to 2000 nm laser is about 16 (Figure 3b). The D* of the device obtained under irradiation at wavelengths ranging from 405 to 2000 nm were all higher than 10^14^ Jones (Figure 3b, see Section S4, Supporting Information, for calculation details), indicating that our 2D BP/G/InSe outperformed almost all previously reported photodetectors based on 2D vdW heterostructure^[^ ^39^, ^40^ ^]^ (see **Table** **1** for comparison). In particular, at excitation wavelength of 532 nm, the D* value reached 3.19 × 10^15^ Jones while at 2000 nm, the D* value reached 3.44 × 10^14^ Jones under room temperature. These values are nearly 2–3 orders of magnitude larger than previous 2D vdW heterojunctions^[^ ^27^, ^41^, ^42^ ^]^ and commercial InGaAs photodetector, whose detectivity is only about 10^12^ Jones when cooled down to 4.2 K. To our knowledge, the D* value of our BP/G/InSe device at 2000 nm is currently the highest for vdW‐heterojunctions‐based photodetectors.

**Table 1:** Comparison of photodetectors with different heterostructure devices

Structures	Type	Measurement conditions	*R*[A W^−1^]	D*[Jones]	Ref.	Year
BP/G^a)^/InSe	Schottky junction	*V*_ds_ = 1 V *V*_g_ = 0 V	3.02 × 10^4^ (532 nm) 3.25 × 10^3^ (2000 nm)	3.19 × 10^15^ (532 nm) 3.44 × 10^14^ (2000 nm)	This work	**–**
BN/MoTe_2_/ G/SnS_2_/BN	Quasi‐PN junction	*V*_ds_ = 1 V *V*_g_ = 0 V	2.69 × 10^3^ (532 nm) 2.63 × 10^3^ (1064 nm) 11.7 (1550 nm)	1.93 × 10^13^ (532 nm) 1.09 × 10^13^ (1064 nm) 1.06 × 10^11^ (1550 nm)	[27]	2019
WSe_2_/G/MoS_2_	Quasi‐PN junction	*V*_ds_ = 1 V *V*_g_ = 0 V	10^4^ (400 nm) <1 (2000 nm)	10^15^ (400 nm) 10^10^ (2000 nm)	[42]	2016
BP/MoS_2_	PN junction	–	0.9 (2500–3500 nm)	1.1 × 10^10^ (3800 nm)	[14]	2018
BP/MoS_2_	PN junction	*V*_ds_ = 3 V *V*_g_ = 0 V	22.3 (532 nm) 0.15 (1550 nm)	3.1 × 10^11^ (532 nm) 2.13 × 10^9^ (1550 nm)	[38]	2016
GaTe/InSe	PN junction	*V*_ds_ = 1 V *V*_g_ = 0 V	267.4 (1064 nm) 1.5 (1550 nm)	≈10^14^ (1064 nm) ≈10^12^ (1550 nm)	[40]	2019
AsP/InSe	PN junction	*V*_ds_ = 2 V *V*_g_ = 0 V	≈1 (520 nm) ≈0.001 (1550 nm)	1 × 10^12^ (520 nm) 2.74 × 10^9^ (1550 nm)	[39]	2019
BP/WSe_2_	Photoconductive	*V*_ds_ = 0.5 V *V*_g_ = 0 V	≈10^3^ (637 nm) ≈0.5 (1550 nm)	≈10^14^ (637 nm) ≈10^10^ (1550 nm)	[25]	2017
BP/InSe	Photoconductive	*V*_ds_ = 1 V *V*_g_ = 0 V	53.8 (655 nm) 43.1 (1550 nm)	–	[43]	2019

^a)^G: few‐layer graphene.

Besides ultrahigh responsivity and specific detectivity, the BP/G/InSe device also has a response speed on the order of ms (Figure 3c and Figure S16, Supporting Information). The response time corresponding to 405, 532, 655, 808, and 2000 nm laser is 336 ms, 334 ms, 367 ms, 881 ms, and 1.20 s, respectively. The relatively slow photoresponse speed of our device may be because of the increased lifetime of the photogenerated carriers,^[^ ^44^, ^45^, ^46^ ^]^ caused by the suppressed interlayer recombination effect in the heterojunction induced by graphene. For further improvement of the photoresponse speed of the device, a new design strategy would be taken like the one recently reported on Schottky photodiode, which achieved an ultrafast photoresponse speed of ≈200 ns.^[^ ^47^ ^]^ Furthermore, when irradiated with visible and NIR light, a photovoltaic effect was observed (Figure S17, Supporting Information). The *R* and EQE of the device without bias voltage is one order of magnitude larger than a previously reported BP/InSe heterojunction^[^ ^26^ ^]^ (Table S1, Supporting Information).

To estimate the probability of interlayer recombination of the BP/G/InSe heterojunction, we analyzed the relationship between photocurrent (*I*_ph_) and light power (*P*_opt_), which can be expressed by a power law:^[^ ^21^ ^]^ *I*_ph_ ≈ *P*_opt_^*α*^ (Figure 3d). The value of *α* under incident light at 405, 532, 655, 808, and 2000 nm was calculated to be 0.503, 0.508, 0.645, 0.596, and 0.138, respectively. The deviation of *α* from 1 corresponds to the loss of photogenerated carriers. The value (0.645) of *α* for *λ* = 655 nm is much larger than the previously reported BP/InSe device,^[^ ^26^ ^]^ whose value is 0.55 for *λ* = 633 nm. These results indicate that insertion of graphene significantly inhibits interlayer recombination of photogenerated carriers at the BP and InSe interfaces, resulting in excellent photoresponse performances when irradiated by laser ranging from 405 to 2000 nm.

**Figure 4:**
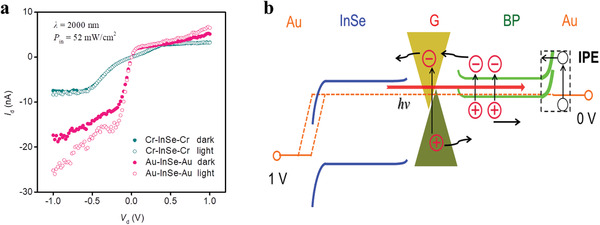
The internal photoemission effect in the BP/G/InSe devices. a) *I*_d_–*V*_d_ curves under 2000 nm laser illumination for the InSe‐FET devices with Au electrodes and Cr electrodes, respectively. b) The light response schematic diagram corresponding to 2000 nm laser excitation.

We find that the IPE^[^ ^48^, ^49^, ^50^ ^]^ effect of the BP/G/InSe Schottky type device also contributes to the ultrahigh D* at 2000 nm. Owing to the relatively large bandgap of InSe (≈1.3 eV), Cr/InSe/Cr devices showed no photoresponse to the 2000 nm laser, whereas Au/InSe/Au devices showed a significant photoresponse (**Figure** **4**a and Figure S18, Supporting Information). This indicates that an IPE effect exists at the interface of the Au electrode and InSe owing to the Schottky barrier. The quasi‐ohmic contact at the Cr/InSe interface, however, did not induce an IPE effect. These results suggest that the Schottky barrier at the Au/InSe interface in the BP/G/InSe device led to the IPE effect, allowing electrons excited by the 2000 nm laser in the Au to pass through BP, G, and InSe and participate in the formation of photocurrents, as shown in Figure 4b. In addition, the schematic diagram shows the photocurrents of the BP/G/InSe device at 2000 nm including photogenerated carriers coming from BP and graphene, and current coming from the IPE effect between Au/InSe interface.

In conclusion, we have demonstrated simple nanostructuring to manipulate the interlayer recombination dynamics of photogenerated carriers at the nanoscale interface between BP and InSe to realize a 2D BP/G/InSe vdW heterostructure with sensitive photodetection performance. Insertion of G altered the transport characteristic of the device from diffusion of minority carriers (p‐n type) to drift motion of majority carrier (Schottky type), contributing to the outstanding photodetection performance. The D* of our device is up to 3.19 × 10^15^ and 3.44 × 10^14^ Jones at the excitation wavelengths of 532 and 2000 nm at room temperature, respectively. Such high photodetection performance is a significant advance over current state‐of‐the‐art 2D heterostructure devices and is particularly promising for sensitive photodetection, especially for the IR range. The efficient structural manipulation of the heterojunction interface by graphene presents a simple way to design future sensitive 2D vdW heterostructure based optoelectronic devices.

## 3 Experimental Section

### Van der Waals Heterostructure Devices Fabrication

The heterostructure devices were fabricated by a vdW adhesion technique to assemble layered materials. First, metal electrodes were attached by electron beam lithography (Raith Pioneer Two) and subsequent deposition (Syskey Technology Co., Ltd., ASB‐EPI‐C6) of 5 nm Cr and 40 nm Au. Then the BP, InSe and graphene flakes were mechanically exfoliated from bulk crystals of BP (SPI Supplies), InSe, and graphite (SPI Supplies), respectively. Then, BP sheet, graphene sheet, and InSe sheet were transferred to the surface of Cr/Au electrodes on a silicon wafer covered by a 300 nm‐thick SiO_2_ layer, respectively, using a viscoelastic stamp (GelFilm by GelPak) and a micromanipulator.

### Atomic Force Microscope

AFM was performed on the Bruker Dimension Icon AFM system in the standard tapping mode.

### Kelvin Probe Force Microscope

KPFM was performed on the Bruker Dimension Icon KPFM system in the standard tapping mode.

### Raman Spectroscopy

Raman scattering was performed on the Horiba Jobin‐Yvon LabRam HR‐VIS high‐resolution confocal Raman microscope equipped with a 633 nm laser as the excitation source at room temperature and a XYZ motorized sample stage controlled by LabSpec software. A 50× objective lens with a numerical aperture of 0. 90 was used to reduce the spot size of the 633 nm laser to about 1 µm.

### Electrical Measurements

The electrical properties of the p‐g‐n devices were tested by a Keithley 4200 semiconductor characteristic analyzer system (Keithley, 4200 SCS) combined with a probe station under ambient conditions.

## Conflict of Interest

The authors declare no conflict of interest.

## Supporting information

Supporting Information

## Data Availability

Research data are not shared.
