# Supplementary material for: Repression of Interlayer Recombination by Graphene Generates a Sensitive Nanostructured 2D vdW Heterostructure Based Photodetector
Source: Adv Sci (Weinh). 2021 May 20;8(15):2100503. doi: 10.1002/advs.202100503 (PMC8336618; doi:10.1002/advs.202100503)
Supplement: Supplementary file 1 — Supporting Information [file ADVS-8-2100503-s001.pdf]

© 2021 Wiley-VCH GmbH

ADVANCED SCIENCE
Open Access

Supporting Information

for *Adv. Sci.,* DOI: 10.1002/advs.202100503

Repression of interlayer recombination by graphene generates a sensitive nanostructured 2d vdW heterostructure based photodetector

Huide Wang, Shan Gao, Feng Zhang, Fanxu Meng, Zhinan Guo, Rui Cao, Yonghong Zeng, Jinlai Zhao, Si Chen, Haiguo Hu, Yu-Jia Zeng, Sung Jin Kim, Dianyuan Fan, Han Zhang,** and *Paras N. Prasad**

Supporting Information

Repression of interlayer recombination by graphene generates a sensitive nanostructured 2d vdW heterostructure based broadband photodetector

Huide Wang, Shan Gao, Feng Zhang, Fanxu Meng, Zhinan Guo, Rui Cao, Yonghong Zeng, Jinlai Zhao, Si Chen, Haiguo Hu, Yu-Jia Zeng, Sung Jin Kim, Dianyuan Fan, Han Zhang,* Paras N. Prasad**

H. D. Wang, S. Gao, F. Zhang, F. X. Meng, Prof. Z. N. Guo, R. Cao, Y. H. Zeng, J. L. Zhao, S. Chen, H. G. Hu, Prof. Y. J. Zeng, Prof. D. Y. Fan, and Prof. H. Zhang
Institute of Microscale Optoelectronics, International Collaborative Laboratory of 2D Materials for Optoelectronics Science and Technology, College of Physics and Optoelectronic Engineering, Guangdong Laboratory of Artificial Intelligence and Digital Economy (SZ),
Shenzhen University,
Shenzhen 518060, China.
E-mail: guozhinan@szu.edu.cn ; hzhang@szu.edu.cn
Prof. S. J. Kim
Department of Electrical and Computer Engineering,
University of Maimi,
Coral Gables, FL 33146, USA.
Prof. P. N. Prasad
Institute for Lasers, Photonics, and Biophotonics and Department of Chemistry,
University at Buffalo, The State University of New York,
Buffalo, NY 14260, USA.
E-mail: pnprasad@buffalo.edu

Supplementary Section 1.

Preparation and characterization of devices

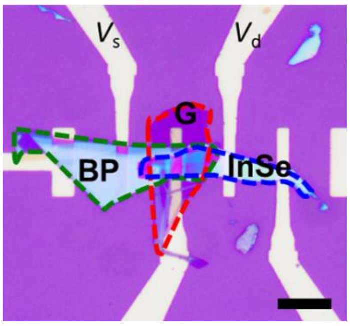

Figure S1. Optical image of the BP/G/InSe photodetector. Scale bar, 10 μm.

Supplementary Section 2.

Principle analysis of the abnormal rectification effect

In order to understand the origin of schottky barrier in heterojunction devices intuitively, the BP/G/InSe device was characterized by kelvin probe force microscope (KPFM). The results show that E_{F_BP} = -4.7 eV, E_{F_G} = -4.85 eV, E_{F_InSe} = -4.8 eV can be obtained if the work function of gold electrode is 5.1eV as a reference.

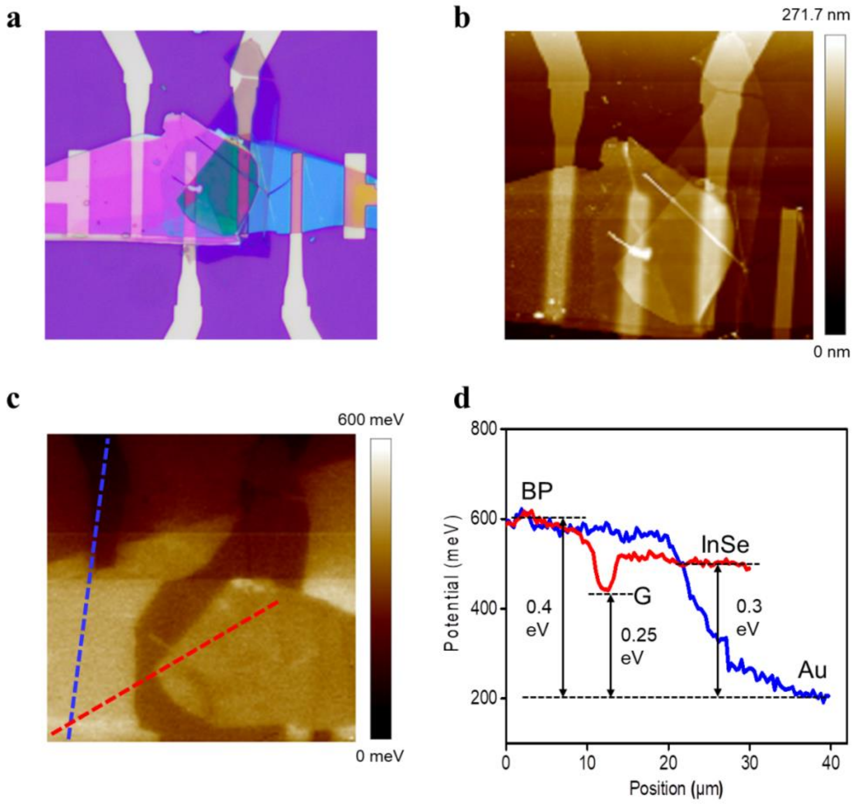

Figure S2. Kelvin probe test of InSe/G/BP heterostructure devices: (a) Optical photo of the device. Scale bar, 10 μm. (b) AFM height diagram of the device. (c) Surface potential diagrams of the device. (d) Surface potential data at positions corresponding to the blue dotted line and the red dotted line in figure (c).

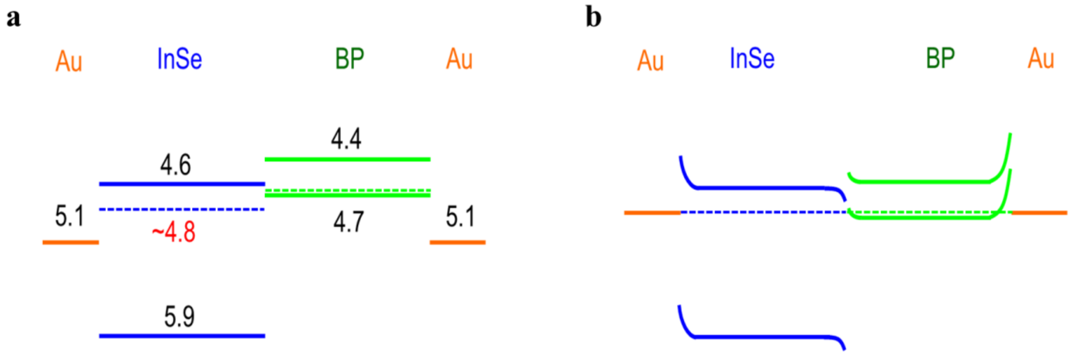

Figure S3. Energy band diagram of InSe and BP before (a) and after (b) contact.

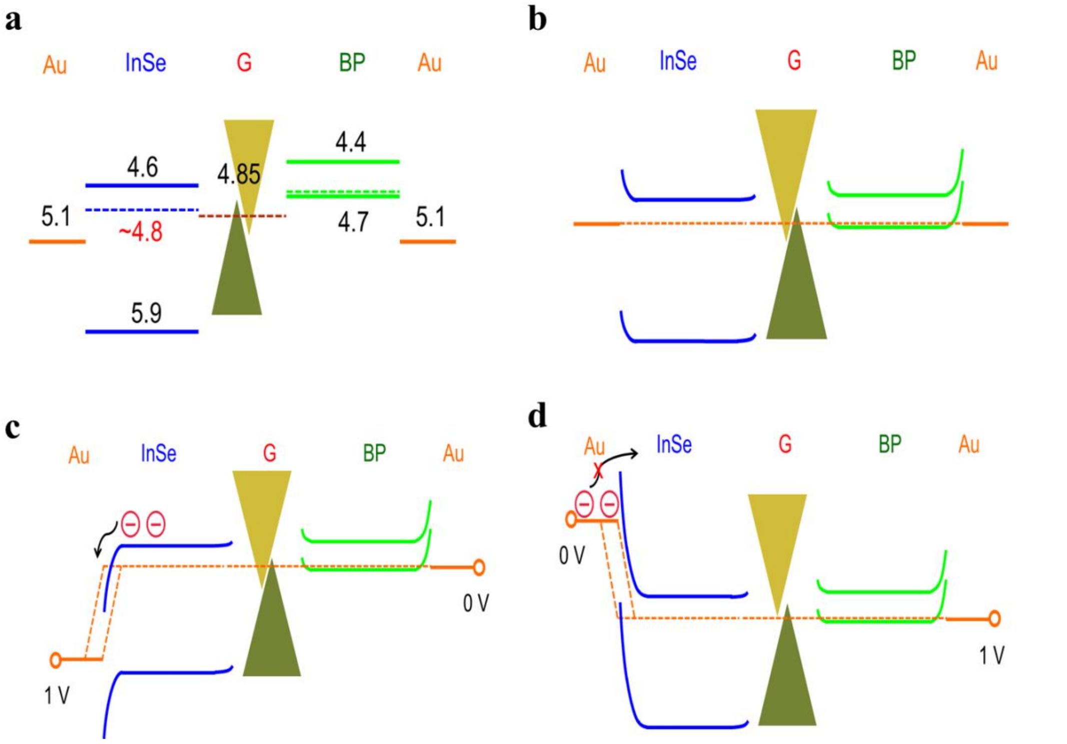

Figure S4. Energy band diagram of BP, G and InSe before (a) and after (b) contact. Band diagram of positive bias voltage (c) and negative bias voltage (d) applied. These analysis indicating that the device has aobvious rectification effect. The bandgap of BP (~26 nm) and InSe sheets (~49 nm) are 0.3 eV and 1.3 eV, respectively.

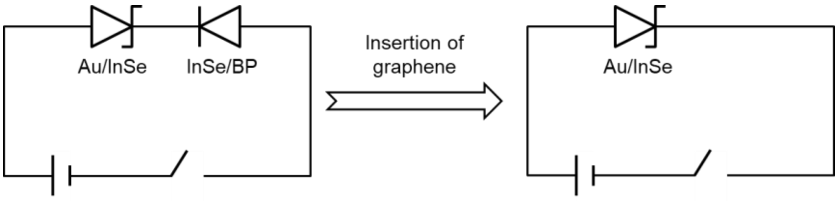

Figure S5. The equivalent circuits of BP/InSe (left) and BP/G/InSe (right).

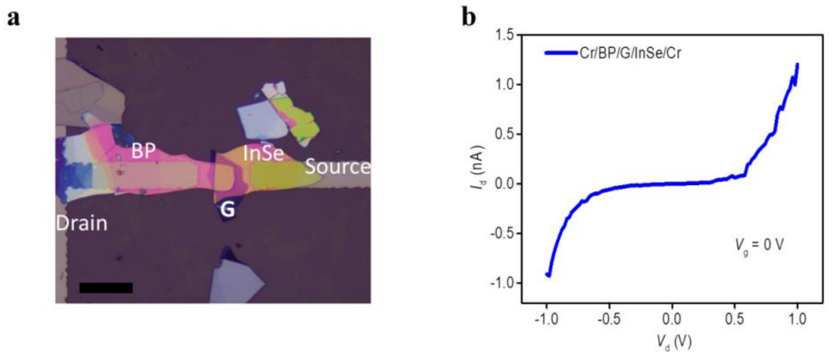

Figure S6. (a) Optical photographs and (b) I-V curves of BP/G/InSe heterostructure devices whose electrodes material is chromium (Cr). The device shows no rectification effect.

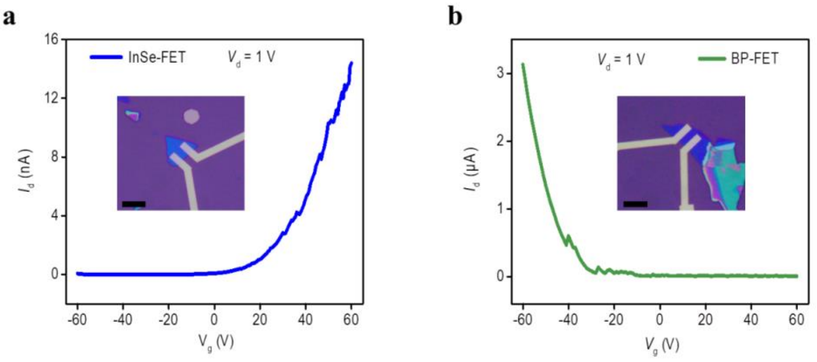

Figure S7. I_{ds} -V_g curves of the (a) InSe-FET and (b) BP-FET on 300 nm SiO_2/Si substrate. The insets show the optical images of the corresponding devices, respectively. Scale bar, 10 μm. For the InSe FET device, I_{ds} increases with increasing positive V_g which indicates a n-type nature. Instead, the BP sheet presents an p-type feature.

Supplementary Section 3.

Characterization of transient absorption spectrum

a, Schematic diagram and test parameters of transient absorption test

Fig. S7 shows the schematic diagram of transient absorption microspectroscopy, in which the pump laser (500 Hz, 400 nm) is generated from the optical parametric amplifier (TPR-TOPAS-U, America), and the probe laser, a femtosecond white continuum (1 kHz, 400 nm–720 nm), is generated by delivering the amplified fs pulses into a thin Ti:sapphire crystal through self-phase modulation (SPM).

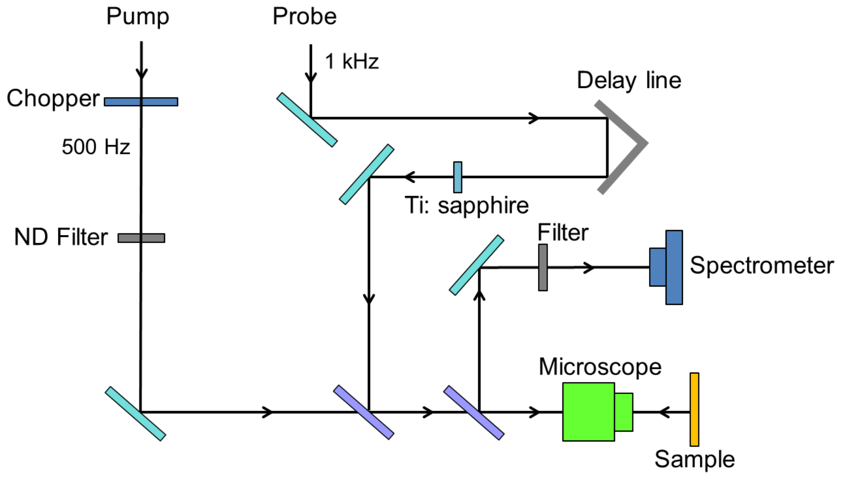

Figure S8. Schematic diagram of the transient absorption experiment.

b, Optical images and test results of heterojunction devices

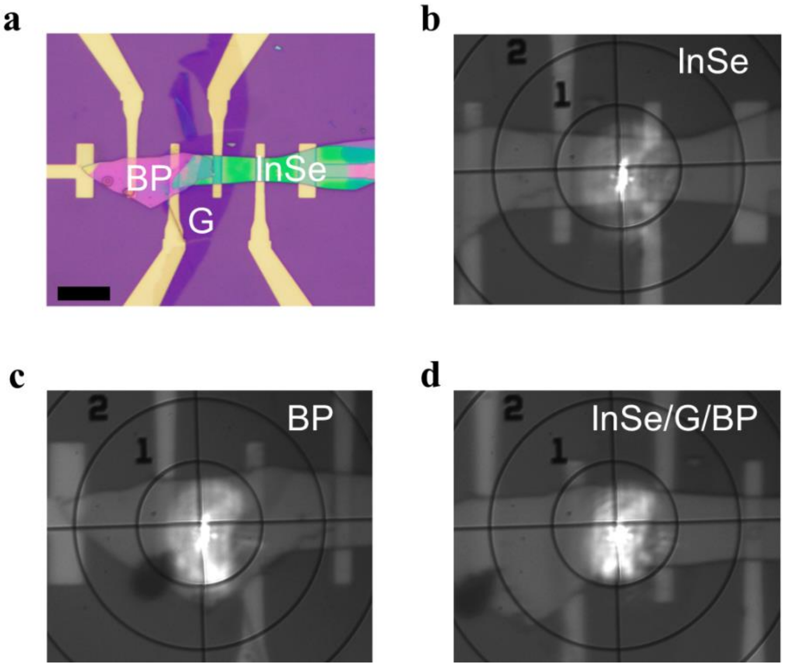

Figure S9. (a) Optical photographs of InSe/G/BP heterostructure device; Optical photographs of (b) InSe, (c) BP and (d) InSe/G/BP heterostructure during transient absorption test, respectively.

Supplementary Section 4.

Photoresponse characteristics of heterojunction devices

a, Photoresponse of heterojunction devices to laser with different wavelengths

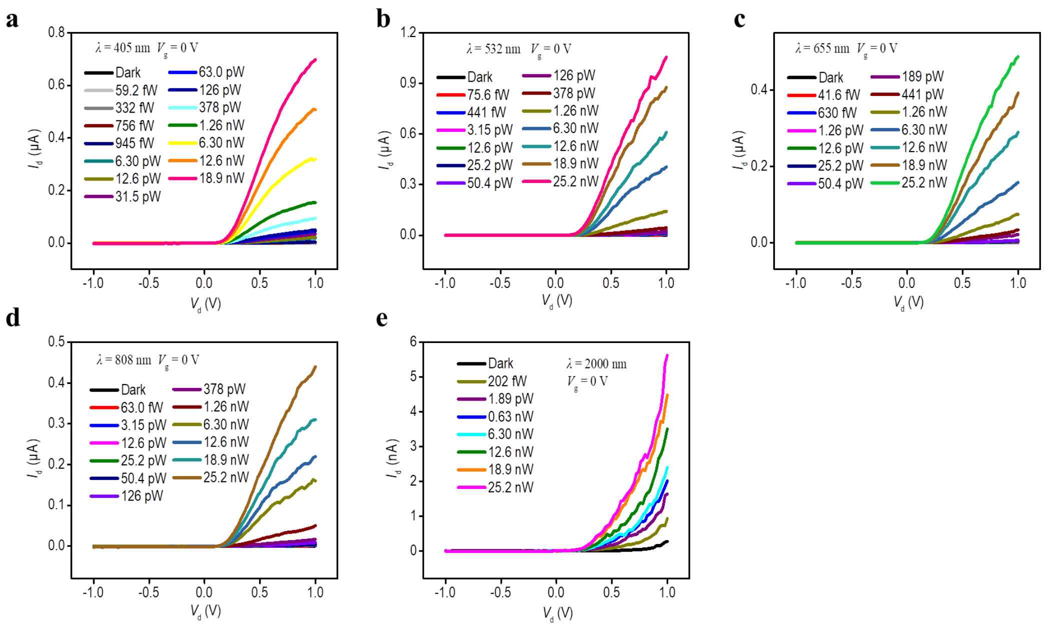

Figure S10. *I-V* curves at different laser power under different wavelength laser illumination of (a) 405 nm, (b) 532 nm, (c) 655 nm, (d) 808 nm and (e) 2000 nm, respectively.

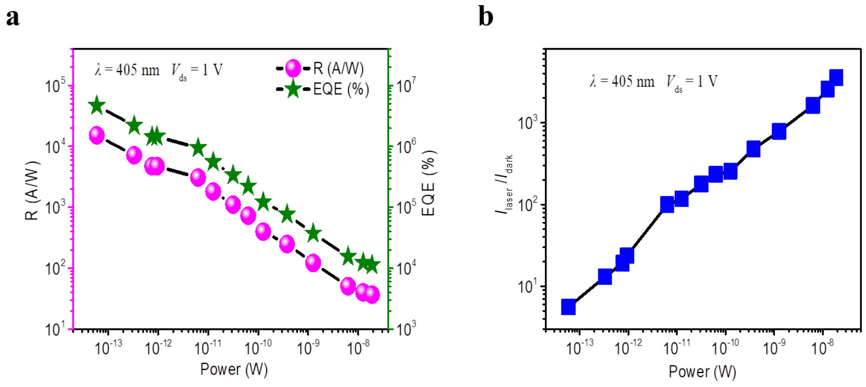

Figure S11. (a) Laser power dependent R and EQE curves under 405 nm laser illumination at V_{ds} = 1 V. (b) Laser power dependent I_{laser}/I_{dark} ratio curve under 405 nm laser illumination at V_{ds} = 1 V.

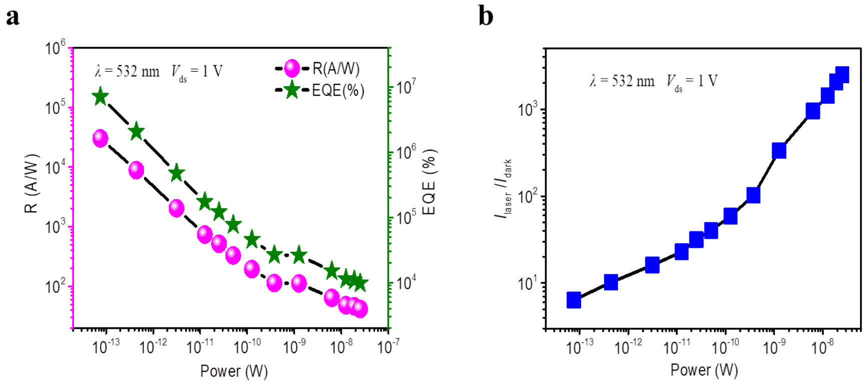

Figure S12. (a) Laser power dependent R and EQE curves under 532 nm laser illumination at V_{ds} = 1 V. (b) Laser power dependent I_{laser}/I_{dark} ratio curve under 532 nm laser illumination at V_{ds} = 1 V.

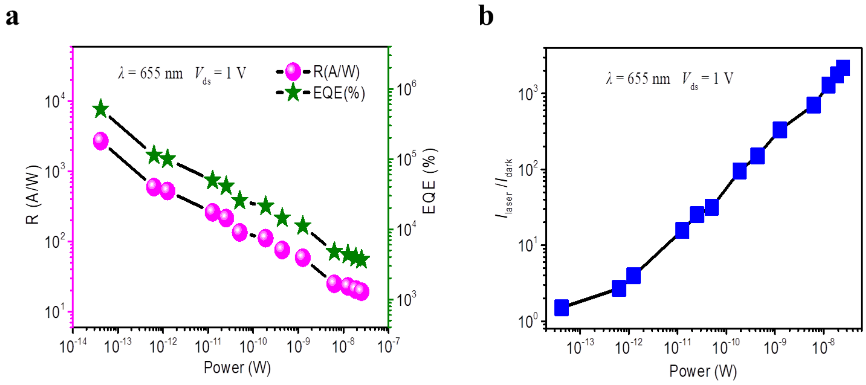

Figure S13. (a) Laser power dependent R and EQE curves under 655 nm laser illumination at V_{ds} = 1 V. (b) Laser power dependent I_{laser}/I_{dark} ratio curve under 655 nm laser illumination at V_{ds} = 1 V.

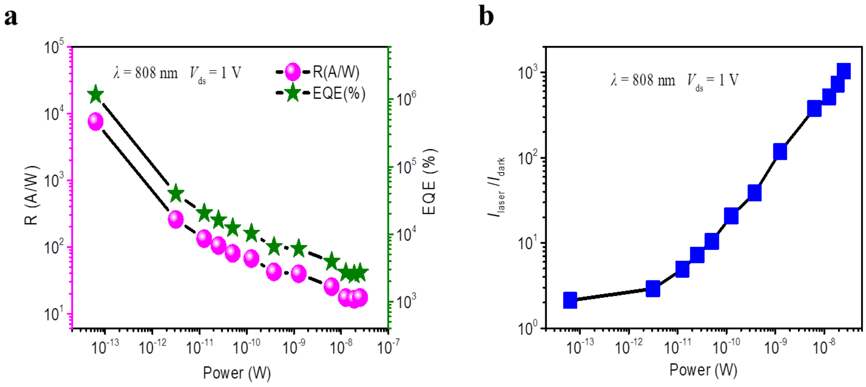

Figure S14. (a) Laser power dependent R and EQE curves under 808 nm laser illumination at V_{ds} = 1 V. (b) Laser power dependent I_{laser}/I_{dark} ratio curve under 808 nm laser illumination at V_{ds} = 1 V.

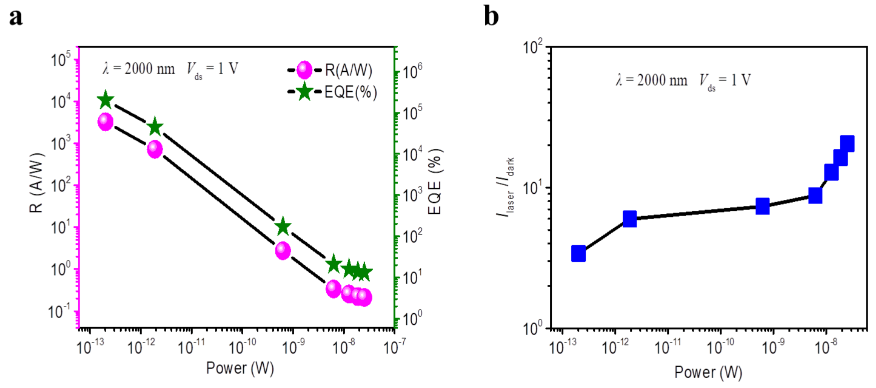

Figure S15. (a) Laser power dependent R and EQE curves under 2000 nm laser illumination at V_{ds} = 1 V. (b) Laser power dependent I_{laser}/I_{dark} ratio curve under 2000 nm laser illumination at V_{ds} = 1 V.

b, Time-dependent current curves with and without laser

We test the current-time curve of the device with alternating laser on and off. The alternating state of laser on and off was controlled by the photoelectric shutter (Thorlabs, SH05), and the duration of the open state and the closed state is all set to 10 s.

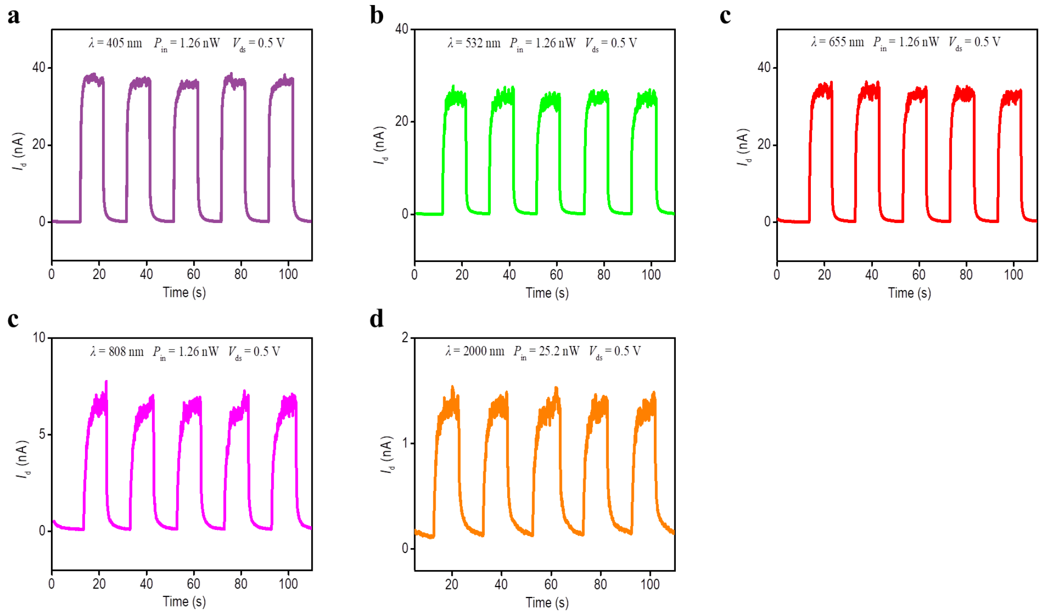

Figure S16. Time-dependent current curves with and without laser at laser wavelength of (a) 405 nm, (b) 532 nm, (c) 655 nm, (d) 808 nm, (e) 2000 nm, respectively.

c, Photovoltaic effects in the photoresponse of heterojunction devices to laser with different wavelengths

We find that the heterojunction devices have a significant photovoltaic effect from Figure S17. The corresponding photovoltaic effect of 405 nm, 532 nm, 655 nm and 808 nm is obvious; The corresponding photovoltaic effect effect of 2000 nm is not obvious, which may be related to the relatively small photocurrent corresponding to 2000 nm laser. The existence of photovoltaic effect also explains the formation of heterostructure.

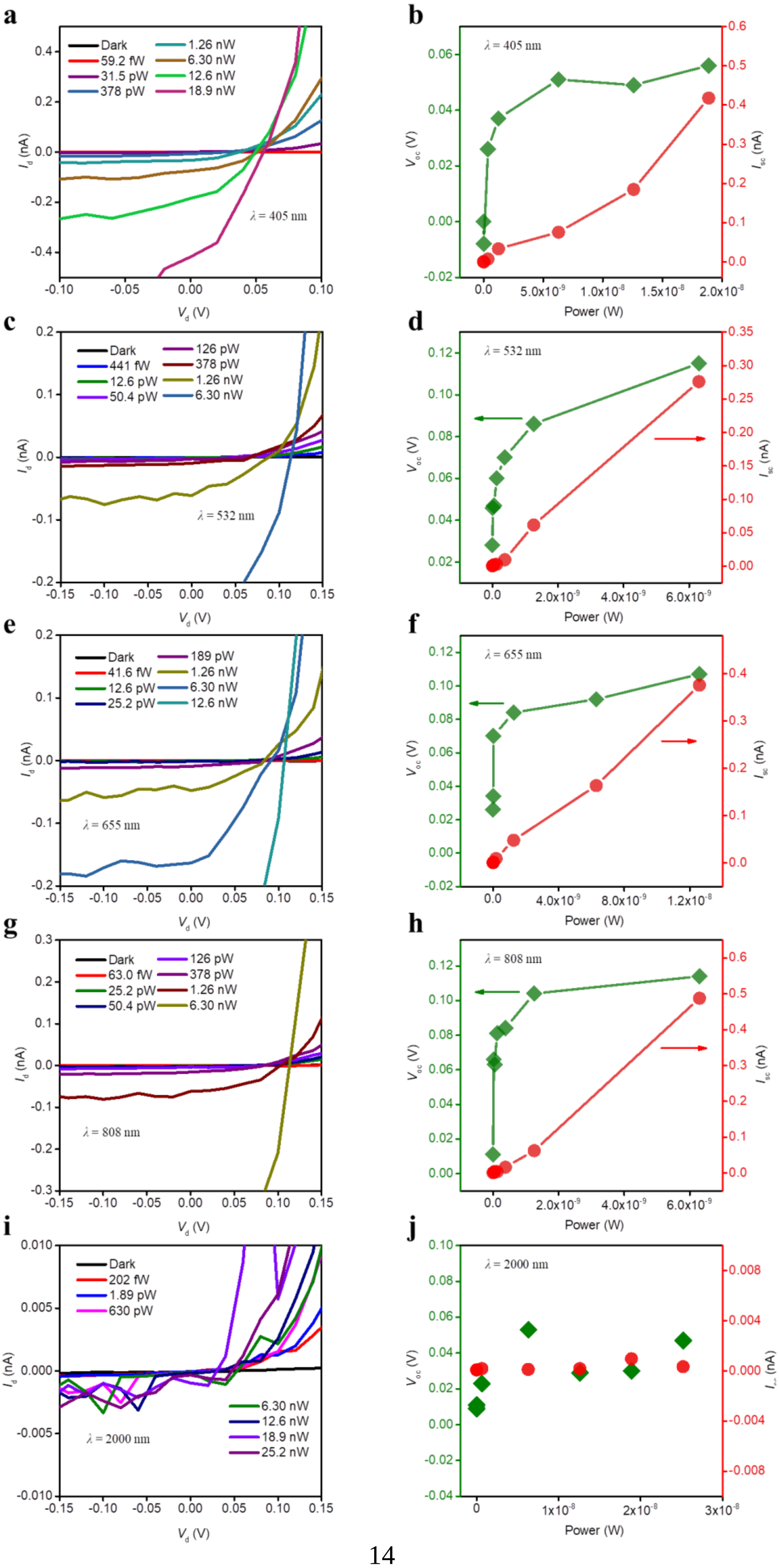
a
Dark
59.2 fW
31.5 pW
378 pW
1.26 nW
6.30 nW
12.6 nW
18.9 nW
λ = 405 nm
I_d (nA)
V_d (V)
b
λ = 405 nm
V_{oc} (V)
I_{sc} (nA)
Power (W)
c
Dark
441 fW
12.6 pW
50.4 pW
126 pW
378 pW
1.26 nW
6.30 nW
λ = 532 nm
I_d (nA)
V_d (V)
d
λ = 532 nm
V_{oc} (V)
I_{sc} (nA)
Power (W)
e
Dark
41.6 fW
12.6 pW
25.2 pW
189 pW
1.26 nW
6.30 nW
12.6 nW
λ = 655 nm
I_d (nA)
V_d (V)
f
λ = 655 nm
V_{oc} (V)
I_{sc} (nA)
Power (W)
g
Dark
63.0 fW
25.2 pW
50.4 pW
126 pW
378 pW
1.26 nW
6.30 nW
λ = 808 nm
I_d (nA)
V_d (V)
h
λ = 808 nm
V_{oc} (V)
I_{sc} (nA)
Power (W)
i
Dark
202 fW
1.89 pW
630 pW
6.30 nW
12.6 nW
18.9 nW
25.2 nW
λ = 2000 nm
I_d (nA)
V_d (V)
j
λ = 2000 nm
V_{oc} (V)
I_{sc} (nA)
Power (W)

Figure S17. Photovoltaic effect in the optical response of devices. *I*−*V* curves of the devices at low voltages at an excitation wavelength of (a) 405 nm, (c) 532 nm, (e) 655 nm, (g) 808 nm, (i) 2000 nm, respectively. Extracted open-circuit voltage (V_{oc}, left axis) and short-circuit current (I_{sc}, right axis) as functions of laser power with excitation wavelengths at (b) 405 nm, (d) 532 nm, (f) 655 nm, (h) 808 nm, (j) 2000 nm.

Table S1. R and EQE of the BP/G/InSe devices under different wavelengths laser at V_{ds} = 0 V.

**Wavelength (nm)**	P_{device} **(nW)**	I_{sc} **(A)**	***R*** **(mA/W)**	***EQE*** **(%)**
405	1.26	3.28×10^{-11}	26.02	7.97
532	1.26	6.13×10^{-11}	48.62	11.33
655	1.26	4.77×10^{-11}	37.83	7.16
808	1.26	6.17×10^{-11}	48.97	7.52

d, Calculation

The R can be calculated by R = I_{ph} / P_{in}, where I_{ph} is the photocurrent under incident illumination, P_{in} is the incident light power received by the actual device with unit of W. The EQE can be calculated by EQE = (hcR)/($e\lambda$), where h is Plank's constant, c is the speed at which light travels in a vacuum, e is the electron charge, and λ is the wavelength of incident light. The D* is measured in units of Jones (cm $Hz^{1/2}$ W^{-1}), which can be calculated by the equation: $D^* = R\ A^{1/2} / (2e\ I_{dark})^{1/2}$, where R is responsivity, A (49.5 μm^2) is the area of the photodetector channel, e is the electron charge, and I_{dark} is the dark current.

e, Photoresponse characteristic of InSe based devices with Au

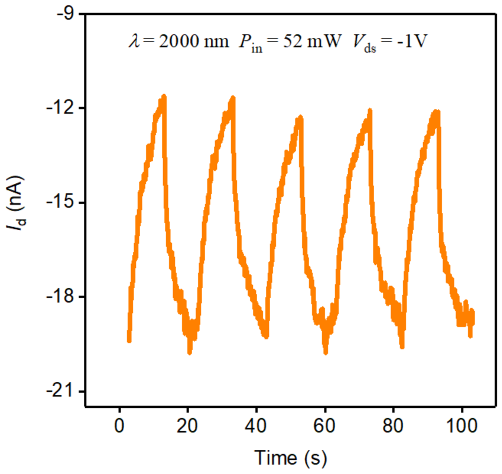

Figure S18. Time-dependent current curve with and without laser at laser wavelength of 2000 nm.
